# Modified pedicle screw–rod fixation as a minimally invasive treatment for anterior pelvic ring injuries: an initial case series

**DOI:** 10.1186/s13018-017-0590-3

**Published:** 2017-06-06

**Authors:** Xiaotian Wu, Zuoqing Liu, Wenqin Fu, Shan Zhao, Juntao Feng

**Affiliations:** 1 Department of Orthopedics, Qingpu Branch of Zhongshan Hospital, Fudan University, Shanghai, 201700 China; 2 Department of Orthopedics, Shuguang Hospital, Shanghai University of Traditional Chinese Medicine, Shanghai, 201203 China

**Keywords:** Pelvic ring, Fractures, Internal fixation, Minimally invasive

## Abstract

**Background:**

Unstable pelvic ring injuries often involve high mortality and morbidity. This study was aimed to evaluate the modified minimally invasive pedicle screw–rod fixation for anterior pelvic ring injuries, in the respects of its feasibility, merits, and limitations.

**Methods:**

Twenty-three patients with unstable pelvic ring injuries underwent the modified anterior pedicle screw–rod fixation, with or without posterior fixation. The clinical outcomes were assessed using Majeed scores, and the quality of reduction was evaluated according to the criteria of Matta.

**Results:**

Majeed scores showed that the clinical outcomes at postoperatively 1 year were excellent in 14 patients, good in 7, and fair in 2. One woman complained of persistent pain at the pubic tubercle during sexual intercourse. Iatrogenic neuropraxia of the unilateral lateral femoral cutaneous nerve occurred in 3 patients. Unilateral femoral nerve palsy occurred in 1 patient. The reduction was found to be excellent in 12 patients, good in 8, and fair in 3. Heterotopic ossification occurred in 8 patients, all being asymptomatic.

**Conclusions:**

The modified pedicle screw–rod fixation with the minimally invasive technique offered an effective alternative for unstable anterior pelvic ring injuries.

## Background

Pelvic fractures represent a relatively small rare injury, but high-energy pelvic ring injuries often involve high mortality and morbidity [1]. Anterior pelvic external fixation is helpful for initial hemodynamic stabilization with less operating time and blood loss than open plating. However, external fixation incurred such complications as tract infection, aseptic loosening, restricted activities, and nerve damage [2]. Hence, successful treatment of unstable pelvic ring injuries remains a challenge for orthopedic surgeons.

Up to now, minimally invasive techniques have been widely used for anterior pelvic ring fixation, whose potential benefits may include reduced blood loss, fewer soft tissue infections, better pain control, and faster rehabilitation. These procedures comprise subcutaneous implants fixed into the ilium with or without fixation into the parasymphyseal region, which have been reported as the pelvic bridge [3, 4] or INFIX [2, 5, 6] technique. Referring to the pelvic bridge technique, we modified routine INFIX for anterior ring fixation in clinical practice, and the pedicle screws and rod were applied for posterior fixation if necessary. This study was aimed to evaluate the modified pedicle screw–rod fixation as a minimally invasive procedure for unstable pelvic ring injuries, in the respects of its feasibility, merits, and limitations.

## Methods

The study was approved by the Ethics Committee of Zhongshan Hospital, and all patients signed informed consent. All procedures involving human participants were in accordance with the Declaration of Helsinki. From January 2013 to October 2015, a total of 23 patients with unstable pelvic ring injuries underwent anterior fixation by the modified INFIX, with or without posterior fixation, which was indicated for unstable anterior ring injuries, especially comminuted fractures. Exclusion criteria included hemodynamically unstable patients, infections or soft tissue defects, acetabular or supra-acetabular fractures, pubic diastases, and a history of pelvic injuries. For posterior fixation, indications were sacroiliac displacement and sacral fractures. The comminuted sacral fractures are the best indications, as no compression is possible for sacroiliac screws. The patients included were 13 men and 10 women with an average age of 36.3 years (range, 20–57 years) and 18 of type B (3 type B1, 7 type B2, and 8 type B3) and 5 of type C (5 type C1) by the Tile classification [7]. Of those patients, 10 were involved in traffic accidents, 7 falls from a height, and 6 from crushes. Preoperatively, all patients received anteroposterior, inlet, and outlet pelvic radiographs and 3-D pelvic CT scans for a full evaluation of the displaced pelvic ring and ipsilateral skeletal or skin traction for fracture reduction. The surgery was scheduled as early as possible when the patient’s physiological condition was stable with an average duration of 4.7 days (range, 1–12 days) from injury to operation.

### Surgical procedures

If posterior instability existed, it is addressed first fixing with the pedicle screws and rod for reduction and fixation. The prone position was employed. We marked the posterior superior iliac spine (PSIS) and dorsal iliac crest preoperatively. Bilateral 3 cm incisions were made along the PSIS. A pedicle finder was used at the medial side of the dorsal iliac crest and 1 cm cranial to the PSIS to make a bony corridor toward the anterior inferior iliac spine (AIIS). After ensuring the corridor did not penetrate the bony cortex, we inserted two 7-mm-diameter pedicle screws with a length of 50 to 60 mm. Partial resection of the PSIS at the entry point for settling the screw heads was performed to minimize implant prominence and soft tissue irritation. A 6-mm-diameter titanium rod was slid through the subfascial plane to connect two screws. To complete reduction of the posterior pelvic ring, compression, distraction, or even injured leg traction was applied according to the fracture pattern. The screw caps were locked at last to maintain the reduction.

**Fig. 1:**
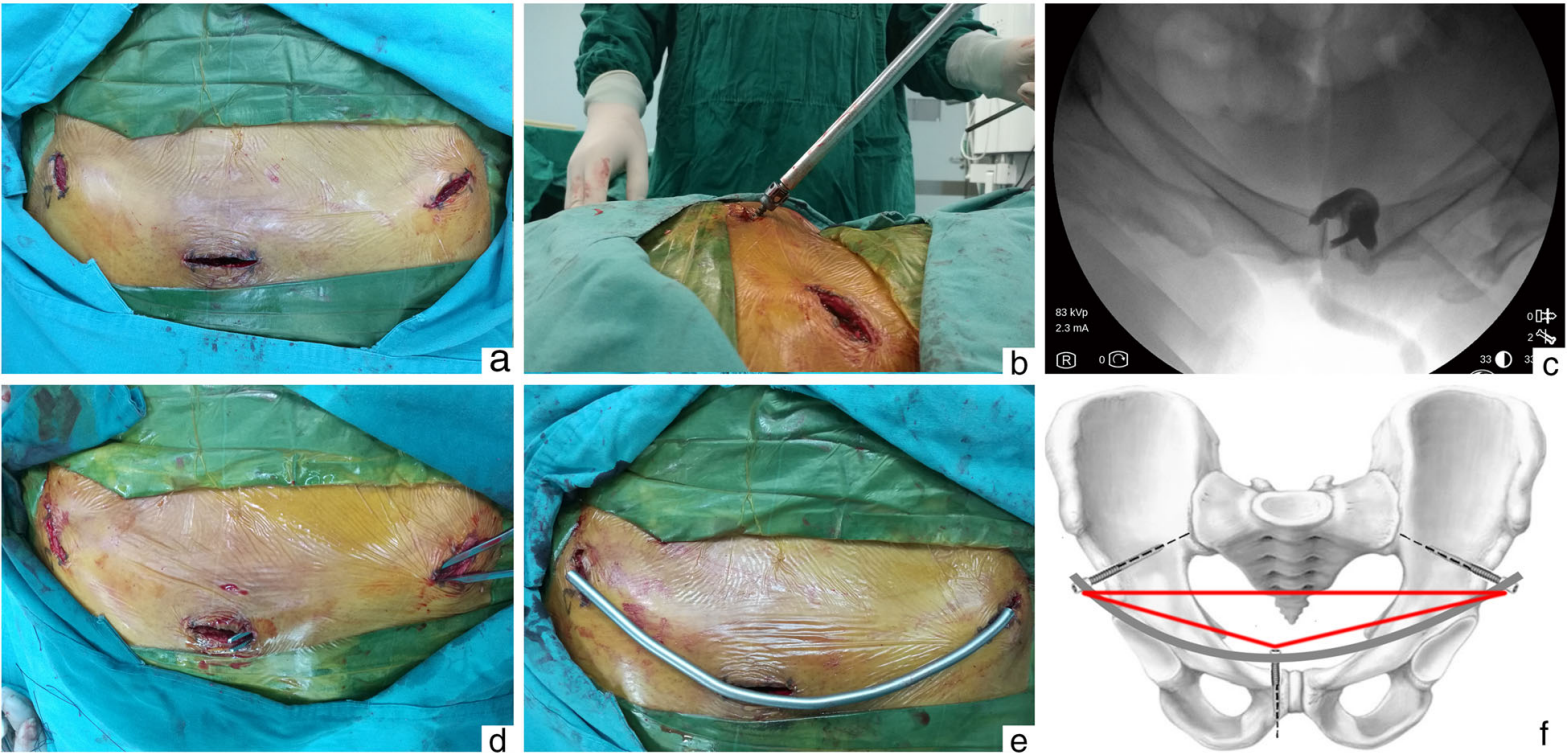
Intraoperative pictures of modified INFIX. **a** Anterior incisions were over both AIIS and the pubic symphysis. **b** The additional screw was inserted at a tilt angle of from 45° to 60°, positioned 2 cm proud of the pubic tubercle. **c** The pelvic inlet view confirmed safe placement of the additional screw. **d** A subcutaneous corridor was developed from the incisions at bilateral AIIS to the Pfannenstiel incision by long hemostats. **e** Schematic diagram of rod placement. **f** Three screws at both AIIS and the pubic tubercle forming a stable geometric triangle

After stabilizing the posterior elements, anterior pelvic ring was addressed. The patient was positioned supine on a radiolucent table. As with routine INFIX, the AIIS was located as the osseous entry with the C-arm fluoroscopy. A “teardrop shape” of the AIIS, the safe zone for pedicle screw placement, was obtained on the obturator outlet view. A 3-cm longitudinal incision was then made centered over the AIIS in line with the groin crease, care being taken to protect the lateral femoral cutaneous nerve (LFCN) which often runs across the surgical field. Access to the AIIS was obtained via a blunt dissection through the interval between the sartorius and tensor fascia lata. A pedicle awl was advanced to open the cortex at the starting pointing, the middle of the AIIS or the center of the “teardrop.” A pedicle finder was then positioned to create a bony corridor for the screw toward the PSIS, using the obturator inlet view to ensure that the corridor did not penetrate the ilium and the iliac oblique view to ensure that the pedicle finder was well clear of the hip joint and greater sciatic notch. Into the corridor, a polyaxial pedicle screw was inserted to an adequate depth positioned roughly 2 cm proud of the bone. Considering the habitus of the patients, screws used for this series were 60 to 80 mm in length and 6.5 to 7 mm in diameter. The procedure was repeated for the contralateral hemipelvis. We modified the INFIX by adding a 3-cm Pfannenstiel incision 2 cm over the pubic symphysis, and the blunt dissection was continued to the level of pubic tubercle. A polyaxial pedicle screw was driven through either ipsilateral or contralateral pubic tubercle down into the inferior ramus, using the pelvic inlet view to confirm the safe placement of this additional screw. Based on our experience, the screw was advanced at a tilt angle from 45° to 60°, also positioned 2 cm proud of the pubic tubercle. The screws used were 35 to 50 mm in length and 6.5 to 7 mm in diameter. When the three screws were positioned in the appropriate depth, a 6-mm-diameter long titanium rod was placed over them to adjust its length and curve. The rod was precontoured with an anterior and inferior bow to avoid compressive neurovascular complications. To develop a corridor just superficial to the fascia, long hemostats were inserted into the subcutaneous tissue from the incisions at bilateral AIIS to the Pfannenstiel incision. The rod was gently slid in the corridor from the incision at ipsilateral AIIS to contralateral AIIS via pubic tubercles and was connected to three screw heads, whose caps were loosely secured to maintain the rod in place. At this point, the reduction tools were attached to the pedicle screws at the bilateral AIIS to manipulate both hemipelvis into place. Reduction of the anterior pelvic ring was achieved by compression or distraction of the rod, depending on the fracture pattern. Finally, the screw caps were locked with a torque screwdriver to maintain the reduction, with the screws at bilateral AIIS being tightened prior to the one at the pubic tubercle (Fig. 1).

### Postoperative management and follow-up

Non-weight-bearing functional exercise on function of lower limbs and joints was initiated for all patients from postoperative day 1. It was important for prophylaxis against deep venous thrombosis. The patients with only anterior ring fixation were allowed partial weight-bearing while sitting on the bedside at postoperatively 4 weeks, and it was allowed at postoperatively 6 weeks for the patients with both anterior and posterior ring fixation. Gradually, they were allowed to walk with full weight-bearing when the union occurred as indicated by callus formation shown on postoperative radiograph. The implant was removed at an average of 7 months postoperatively (range, 4–10 months). Routine follow-up were arranged at postoperatively 4, 6, 3, 6, and 9 months and 1 year for clinical and radiological examinations. Follow-up was continued after this period if necessary. At the last follow-up, the outcome was assessed clinically (Majeed scores) [8] with five functional indicators: pain, sitting, standing, sexual intercourse, and work. Postoperative reduction was evaluated according to the criteria of Matta [9], graded by displacement as excellent (0–4 mm), good (5–10 mm), fair (11–20 mm), and poor (>20 mm) (Table 1).

**Table 1:** Patients’ characteristics and outcomes

Gender	Age (years)	Tile type of fracture	Injury mechanism	Time to surgery (days)	Surgical procedures	Operation time (min)	Blood loss (ml)	Reduction for fracture	Majeed scores/grade	Complications
M	23	C1	Traffic injury	6	Modified INFIX + posterior fixation	71	75	Good	74/good	LFCN irritation, heterotopic ossification
M	34	B2	Fall injury	4	Modified INFIX	40	34	Good	93/excellent	Heterotopic ossification
F	29	B1	Traffic injury	5	Modified INFIX	37	33	Excellent	94/excellent	
M	57	C1	Fall injury	10	Modified INFIX + posterior fixation	79	70	Excellent	87/excellent	Heterotopic ossification
F	33	B2	Traffic injury	3	Modified INFIX + posterior fixation	59	66	Good	82/good	
F	37	B3	Crush injury	5	Modified INFIX + posterior fixation	62	53	Fair	76/good	Heterotopic ossification
M	45	B2	Traffic injury	2	Modified INFIX	46	37	Excellent	94/excellent	
F	20	B3	Fall injury	9	Modified INFIX + posterior fixation	69	55	Excellent	91/excellent	
M	38	B1	Crush injury	1	Modified INFIX	34	29	Excellent	95/excellent	
M	42	B2	Crush injury	3	Modified INFIX + posterior fixation	53	42	Excellent	89/excellent	
M	53	B3	Fall injury	5	Modified INFIX + posterior fixation	62	47	Fair	64/fair	Heterotopic ossification
F	29	B3	Traffic injury	3	Modified INFIX + posterior fixation	63	61	Good	79/good	LFCN irritation
F	31	B3	Fall injury	5	Modified INFIX + posterior fixation	59	41	Excellent	89/excellent	
M	49	C1	Crush injury	6	Modified INFIX + posterior fixation	68	58	Good	75/good	Heterotopic ossification
F	43	B3	Traffic injury	4	Modified INFIX + posterior fixation	57	39	Fair	66/fair	Femoral nerve palsy
M	33	B2	Crush injury	4	Modified INFIX	41	25	Excellent	92/excellent	
F	36	C1	Traffic injury	4	Modified INFIX + posterior fixation	63	67	Good	87/excellent	Heterotopic ossification
M	35	B3	Traffic injury	12	Modified INFIX + posterior fixation	74	69	Excellent	93/excellent	
M	43	C1	Fall injury	3	Modified INFIX + posterior fixation	57	45	Good	77/good	Heterotopic ossification
F	45	B2	Traffic injury	4	Modified INFIX + posterior fixation	67	63	Excellent	94/excellent	LFCN irritation
M	30	B1	Crush injury	2	Modified INFIX	30	23	Excellent	96/excellent	
F	25	B2	Fall injury	3	Modified INFIX	33	26	Good	79/good	Pain during sex
M	24	B3	Traffic injury	5	Modified INFIX + posterior fixation	65	58	Excellent	89/excellent	

## Results

**Fig. 2:**
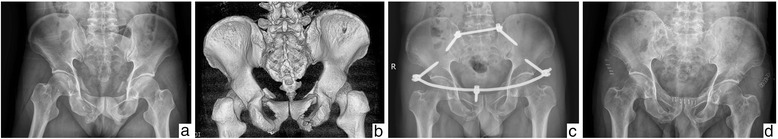
A 35-year-old male patient with anterior and posterior pelvic ring injuries caused by a traffic accident. **a**, **b** Preoperative X-ray plain film and 3-D CT image showing bilateral pubic ramus fracture combined with avulsion fracture of PSIS. **c** Postoperative X-ray plain film showing good reduction with modified anterior INFIX and posterior fixation. **d** X-ray plain film showing bone union as indicated by callus formation at postoperative 7 months

In our series, 7 patients underwent anterior fixation of modified INFIX only, and 16 patients both anterior and posterior fixation. The operation for anterior ring fixation took an average of 37.3 min (range, 30–46 min), with mean intraoperative blood loss of 29.1 ml (range, 23–37 ml) and for both anterior and posterior fixation an average of 64.2 min (range, 53–79 min) with mean intraoperative blood loss of 56.8 ml (range, 39–75 ml). All patients were followed up for an average of 15 months (range, 13–20 months), and no one died or was lost to follow-up. None had postoperative complications of hemorrhagic shock, deep venous thrombosis, or wound infection. Majeed scores showed the clinical outcome at postoperatively 1 year was excellent in 14 patients, good in 7, and fair in 2. All patients could normally sit, stand, squat, and lie prone or on side. The screw caps and the rod could be palpated without sharp pain except in one lean woman who complained of persistent pain at the pubic tubercle during sexual intercourse. The pain was eliminated with the implant removal. Iatrogenic neuropraxia of unilateral LFCN occurred in 3 patients but disappeared spontaneously at postoperatively 1 month. Unilateral femoral nerve palsy was recorded in 1 patient at postoperatively 1 day, who was returned to operation emergently for adjustment of the anterior ring fixation. The symptom was then relieved gradually. This patient fully recovered within 2 months after the removal of the anterior implant. Bone union was achieved for all pelvic fractures without nonunion or malunion. The postoperative radiograph showed no secondary displacement or loosening of or fracture of the implant. Heterotopic ossification at the screw heads was observed in 8 patients, who were all clinically asymptomatic. Evaluation by the criteria of Matta showed the reduction was excellent in 12 patients, good in 8, and fair in 3. Radiographs of a typical case were provided to illustrate the preoperative and postoperative changes (Fig. 2).

## Discussion

The pelvic ring is formed by the ilium, ischium, pubis, and sacrum connected by ligaments. The anterior pelvic ring, accounting for 30% of pelvic stability, consists of pubic symphysis, bilateral pubic ramus, and ventral ilium and the posterior ring, accounting for 70%, mainly of sacrum, dorsal ilium, and sacroiliac joint complex [10]. Vertically unstable pelvic ring injuries require anterior and posterior fixation. Anterior fixation, a complement to posterior fixation, allows stabilization in the horizontal plane and reduces anterior pelvic widening.

First reported by Kuttner et al. in 2009 [11], INFIX is the pedicle screw–rod fixation specifically used for anterior pelvic ring injuries. It involves two supra-acetabular pedicle screws and a subcutaneous interconnecting rod [2, 5, 6]. The type of the pedicle screw determines the performance of INFIX, with monaxial screws providing significantly greater stiffness than polyaxial screws [12]. However, polyaxial screws were reported to be able to reduce the difficulty of rod manipulation. Similarly, the pelvic bridge, pioneered by Cole et al. [3, 4] in 2012, involves a reconstruction plate or a plate-rod system spanning injured anterior pelvic structures between the iliac wing and the pubic tubercle.

Inspired by the pelvic bridge, we modified INFIX. With the Pfannenstiel incision as used in the pelvic bridge, we added a polyaxial screw in either pubic tubercle. Thus, three screws at both AIIS and the pubic tubercle formed a stable geometric triangle which provided extra conjunctive points between the screw and the curved rod. The curvature of the rod fitted better with the anatomy of the anterior pelvic ring. Hence, this modified three-point INFIX frame was thought to be more stable than the routine two-point INFIX frame for anterior pelvic ring injuries. As with the pelvic bridge, no damage was incurred on any fascia or muscle in the Pfannenstiel incision because the screws were directly positioned over the tubercles where the rectus tendons attach [13], which minimized the risk of ilioinguinal and iliohypogastric neuropathy. The additional Pfannenstiel incision was also used to create a subcutaneous corridor to bilateral AIIS as is done in the pelvic bridge for bilateral pubic ramus fractures. In routine INFIX, the subcutaneous corridor is created directly from one side of AIIS to the other with high risk of neurovascular injury and abdominal perforation. Different from routine INFIX, the rod in our modified modality was not placed under the Bikini line but still in the Bikini area, which stays in a relatively stable position from sitting to standing in all individuals [14]. Our study results verified no interference with sitting, standing, squatting, or even lying prone. No sharp pain was caused by screws and the rod, except in a lean woman who complained of persistent pain at the pubic tubercle during sexual intercourse. This was attributed to the irritation by the screw in the pubic tubercle over which the soft tissue was so thin in this woman. The symptom disappeared after the removal of the screw. Both clinical and radiological results showed no selective difference of the additional screw position at either side of the pubic tubercle. According to our experience, the screws at bilateral AIIS should be first secured to ensure that the strain is concentrated in the supra-acetabular region which, with dense cancellous bone, affords excellent purchase for fixation. Although the pubic tubercle is thin in bone composition, the additional screw aided in pullout strain. As reported in Vaidya et al. [2], we gave the priority to posterior stability. Gardner et al. [5], however, suggested performing anterior fixation first for the reduction of both the anterior and posterior pelvis.

Controversy exists as to the appropriate screw size used in routine INFIX. Owen et al. [15] reported failure of fixation with small-diameter screws and salvage with larger screws in the morbidly obese patient, while Hoskins et al. [16] attributed traction-induced neuropraxia to the size of the large screws. In the modified INFIX, we used small-diameter screws without loss of reduction and increased blood loss but with longer operation time (64.2 min) than the routine INFIX (51 min) [16]. LFCN irritation is the most prevalent iatrogenic neurovascular complication for INFIX, and damage of LFCN may be inflicted during the dissection, the rod placement, or even the rod removal. As the irritation is associated with the length of the rod end lateral to the screw, the rod should be trimmed as short as possible to minimize it. Temporary neuropraxia of unilateral LFCN was observed in 13% (3 of 23) patients, lower than 30% reported by Vaidya et al. [17], which might be explained by the smaller-diameter screws we used. The symptoms were all transient and never recurred after the rod removal. Femoral nerve palsy was considered a rare and devastating complication of INFIX, a likely cause being the limited space available for the psoas and femoral nerve [18]. The cadaveric study found the femoral nerve might be the structure most likely at risk of compression by the rod and the compression of the femoral nerve was directly related to the depth of screw insertion [19]. To prevent femoral nerve injuries, careful physical examination of the nerve function both before and after surgery is important. We recommend using polyaxial screws, and more space available between the screw and rectus fascia should be kept during screw insertion. The screws are suggested to be 15–40 mm proud of the bone, depending on the patient’s habitus [2]. Hesse et al. [18] reported that resolution of femoral nerve palsy was variable and incomplete even after the removal of the implants. In our series, only one unilateral femoral nerve palsy was noted. The implant was adjusted emergently and the symptom was relieved gradually, and a full recovery was obtained after the removal of the implant. We believed that early diagnosis and adjustment without delay played a significant role in nerve recovery. Heterotopic ossification at the screw heads was an asymptomatic finding in 34.8% (8 of 23) patients, comparable to 35% with routine INFIX [17]. Thorough rinsing of the surgical site was recommended to avoid this complication.

This was a single-center retrospective clinical series where modified pedicle screw–rod fixation was employed for anterior pelvic ring injuries. Limitations of our study need to be mentioned. First, the small sample size and lack of long-term follow-up warranted future multicenter prospective study for the final evaluation. Second, our analysis was based on clinical cases to predict the stability of fixation technique, and a randomized case–control study is necessary for more convincing conclusions. Third, the study, an indirect deduction in nature, lacked direct biomechanical evidence. In particular, it remains uncertain whether the injured side of the pubic tubercle is different from the intact side in biomechanical stability for the screw position. Fourth, the reduction proved difficult compared to that with classic plate fixation. Thus, acetabular or supra-acetabular fractures, which need excellent anatomical reduction, are the contraindication of our method. Infections or soft tissue defects in surgical area are relative contraindications.

## Conclusions

Although the modified INFIX involved the additional incision and screw, it afforded satisfactory clinical and radiological outcomes with less complication. We believe that the modified technique offers an effective alternative in the treatment of anterior pelvic ring injuries. Indications for its use need to be more clearly ascertained. Future multicenter prospective randomized studies are warranted for more convincing evaluation.

## Abbreviations

AIIS: Anterior inferior iliac spine
CT: Computed tomography
LFCN: Lateral femoral cutaneous nerve
PSIS: Posterior superior iliac spine
